# Polarity Inversion of Aluminum Nitride Thin Films by using Si and MgSi Dopants

**DOI:** 10.1038/s41598-020-61285-8

**Published:** 2020-03-09

**Authors:** Sri Ayu Anggraini, Masato Uehara, Kenji Hirata, Hiroshi Yamada, Morito Akiyama

**Affiliations:** Advanced Manufacturing Research Institute (AMRI), National Institute of Advanced Industrial Science and Technology (AIST), 807-1 Shukumachi, Tosu, Saga 841-0052 Japan

**Keywords:** Chemistry, Materials science

## Abstract

Polarity is among the critical characteristics that could governs the functionality of piezoelectric materials. In this study, the polarity of aluminum nitride (AlN) thin films was inverted from Al-polar to N-polar by doping Si into AlN in the range of 1–15 at.%. Polarity inversion from Al-polar to N-polar also occurred when MgSi was codoped into AlN with Mg to Si ratio was less than 1. However, the polarity can be reversed from N-polar to Al-polar when the ratio of Mg and Si was greater than 1. The effect of Si and MgSi addition was investigated with regards to their crystal structure, lattice parameters, polarity distribution and the oxidation state of each elements. Furthermore, the effect of intermediate layer as well as the presence of point defect (i.e. aluminum vacancy) were investigated and how these factors influence the polarity of the thin films are discussed in this report.

## Introduction

Wurtzite structure aluminum nitride (AlN) is one of the important building blocks for various advanced electromechanical and optoelectronics devices. AlN has been touted as a promising material for radio frequency (RF) acoustic devices such as resonators, due to its high acoustic velocity, high quality (Q) factor, thermal stability and the compatibility with CMOS technology^1^. With the ever-growing market of wireless telecommunication devices, the need to continuously enhance the piezoelectric property of AlN has made it the subject of extensive study and research.

Improving the piezoelectric properties of AlN can be done by either augmenting the magnitude of piezoelectric response along c-axis (*d*_33_) or by controlling the polarity of the thin film. Since the non-centrosymmetry of wurtzite AlN is the origin of polarization along the c-axis, a highly *c*-oriented AlN thin film could exhibits either Al- or N-polarity^2^. While considerable efforts have been devoted to find the best dopants that could produce the highest enhancement in the piezoelectric response^3–5^, manipulating the polarity of AlN is equally essential in designing high performance devices, since having thin film with different polarity could lead to different electronic property and eventually alter the performance of the developed device^2,6–8^. For example, having a stack of N-polar layer on top of an Al-polar layer enabled a solidly mounted resonator BAW (SMR-BAW) to operate at higher frequency which made it capable to function in more advance telecommunication technology^2,6^.

When it comes to controlling the polarity, there are numbers of efforts that have been reported to successfully switch the polarity of nitride thin films from metal-polar into N-polar or vice versa^1,8–12^. The insertion of a buffer layer or an intermediate layer is one of the common approaches that was utilized to inverse the polarity^8–10,13,14^. Aside from this, switching the polarity can also be done by altering the thin film deposition parameters (e.g. pressure or target power), inserting metal seed or by introducing oxygen during thin film deposition^12,15–17^. However, these methods are reported to cause deterioration in crystallinity as well as the piezoelectric properties^6^, which was why Mizuno *et al*. introduced the use Germanium (Ge) as a dopant to inverse the polarity of AlN from Al- to N-polar without deteriorating the piezoelectricity^6^. Since Si is in the same V-group with Ge and more economical than Ge, we proposed the use of Si-based dopants to control the polarity of AlN-based thin films. In addition to investigating the use of Si as a single dopant, MgSi was also codoped into AlN and the piezoelectric response (*d*_33_) as well as the polarity are examined. The motivation behind the use of MgSi was based on the fact that several Mg-based codopants are reported to capable of enhancing the piezoelectric property of AlN^3,4,18^. Thus, addition of MgSi into AlN here was intended to improve the *d*_33_ of AlN. In this study, the effect of Si and MgSi dopants on crystal structure, lattice parameters, chemical state of each elements were investigated. In order to elucidate the polarity inversion, the effect of intermediate layer on polarity inversion was examined and the presence of point defect (vacancy) was also verified. Based on the obtained results, a possible mechanism for polarity inversion is also proposed herein.

## Results & Discussion

### Effect of Si addition as single dopant on the piezoelectric response and the polarity

**Figure 1:**
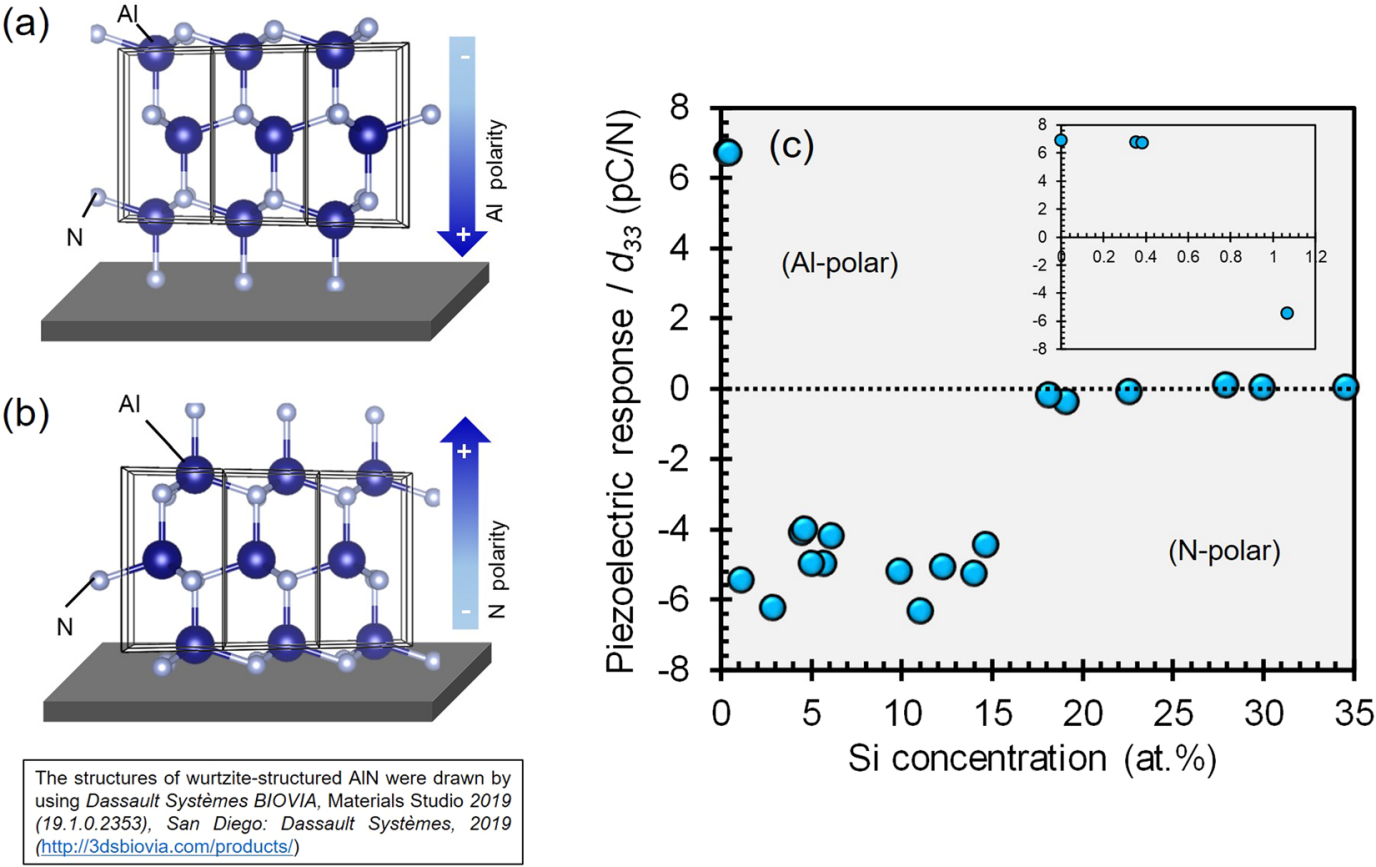
Illustration of thin film with (**a**) Al-polar or (**b**) N-polar as well as (**c**) the effect of Si addition on piezoelectric response of Si_*x*_Al_1−*x*_N thin films (inset of Fig. 1(c) is the effect of Si addition on piezoelectric response of Si_*x*_Al_1−*x*_N with *x* < 1.2).

In this study, positive piezoelectric responses (*d*_33_) indicate that the polarity of AlN-based thin film is predominantly oriented toward the substrate (Al-polar) (Fig. 1(a)), while negative *d*_33_ suggests that the thin film is oriented in the opposite direction (N-polar) (Fig. 1(b)). As shown in inset of Fig. 1(c), the magnitude of *d*_33_ is found to be unaffected by the addition of Si in lower concentration range (<1 at.%) and the positive *d*_33_ value suggests that the polarity of these thin films is mainly comprised of Al-polar (inset of Fig. 1(c)). Meanwhile, the negative *d*_33_ (−4 to −6.3 pC/N) that is observed upon introduction of 1–15 at.% Si into AlN indicates that the polarity of the thin films is predominantly N-polar. However, the *d*_33_ gradually decreases as the concentration of Si is greater than 15 at.% (Fig. 1(c)).

**Figure 2:**
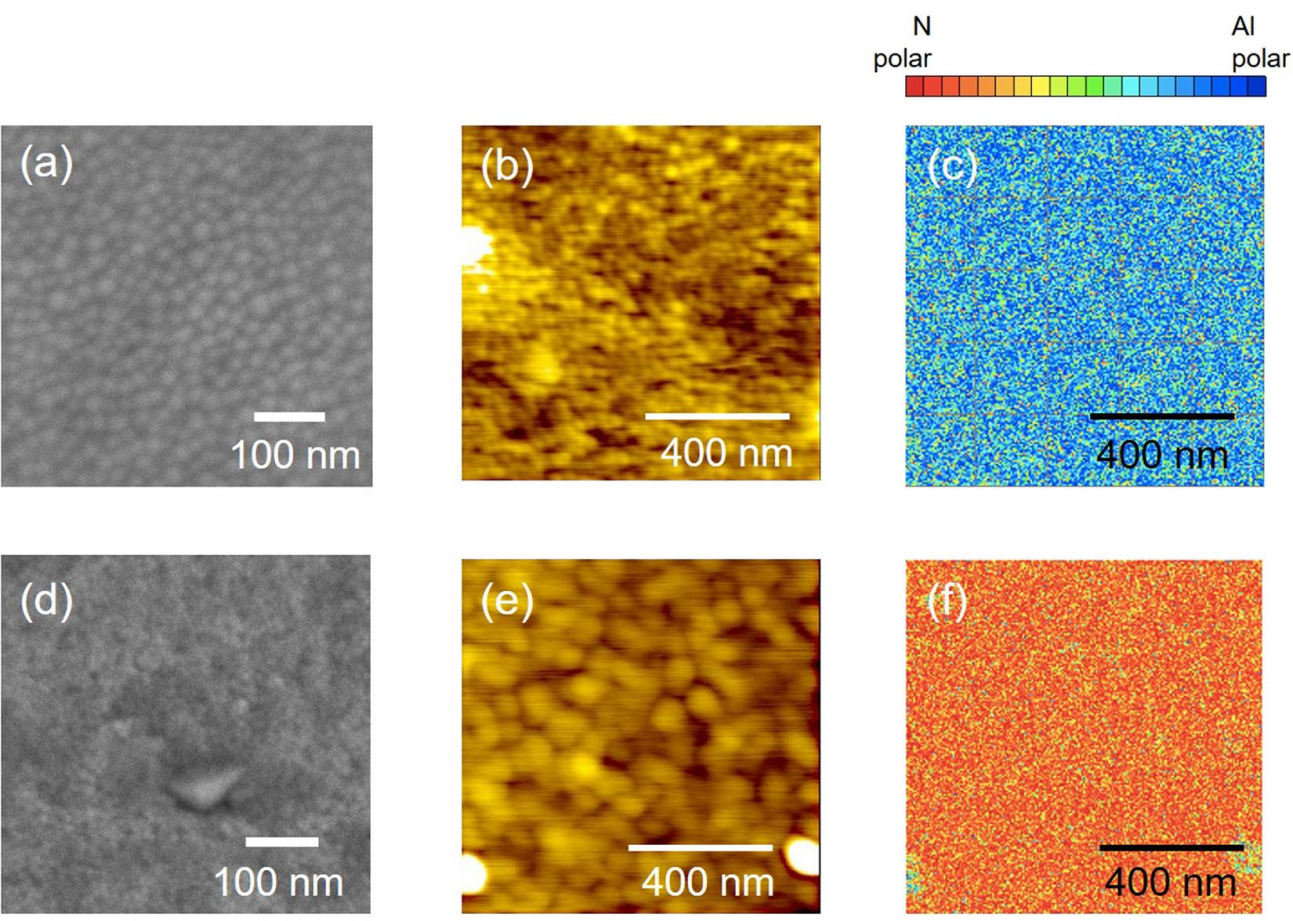
SEM, AFM and PFM images of AlN (**a–c**) and Si_0.11_Al_0.89_N (**d–f**).

The surface morphology of the thin films was investigated via scanning electron microscopy (SEM) and atomic force microscopy (AFM) while their polarity distribution was studied by piezoresponse force microscopy (PFM) measurements. For this investigation, Si_0.11_Al_0.89_N which has been confirmed to generate negative *d*_33_ (−6.3 pC/N) is chosen as the representative of Si_x_Al_1−X_N thin films that exhibits N-polar. As can be seen in Fig. 2(a–c), AlN is found to consist of particles with size in the range of 15 to 35 nm and is predominantly composed of Al-polar component. However, the particles of Si_0.11_Al_0.89_N in the range of 35–70 nm and the thin film is mainly comprised of N-polar components (Fig. 2(d–f)). The polarity distribution for these samples is in good agreement with that observed in Fig. 1(c).

### Effect of Si addition as single dopant on the crystal structure

**Figure 3:**
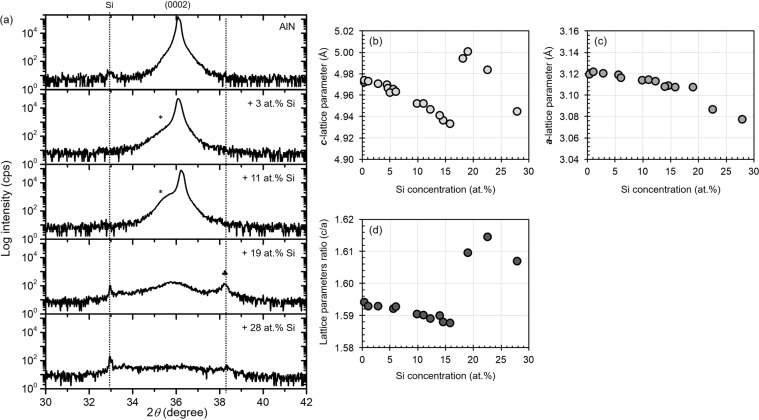
Effect of Si addition into AlN on (**a**) (0002) peaks and the corresponding lattice parameters ((**b**) *c*-axis and (**c**) *a*-axis)) as well as the (**d**) lattice parameters ratio (*c/a*).

Since changes in piezoelectricity of AlN usually correlates with changes in wurtzite structure and its lattice parameters^4,5^, effect of Si addition on the wurtzite structure and the corresponding lattice parameters were examined. As shown in Fig. 3(a), the (0002) of wurtzite structure that is normally observed at 36° is found to shift to lower degree for thin films with Si addition <19 at.%. However, unknown peak (*) at 35.5° is also observed as a shoulder peak of (0002) for Si-doped-AlN (Si <19 at.%), which indicates the presence of additional phase that accompanied wurtzite-structured compound. However, since the appearance of peak at 35.5 ° is also a characterization of zinc blende AlN (3C-AlN)^19^, there is a possibility that this shoulder peak might be an indication of 3C-AlN. At greater Si additions (Si ≥ 19 at.%), the intensity of (0002) is significantly decrease and followed by the appearance of additional unknown peak at 38.3° (♣). Peaks that are observed in the in-plane x-ray diffraction (XRD) profile for the examined Si_x_Al_1−x_N thin films are in good agreement with wurtzite AlN (ICSD no. 34236) (Supplementary 1) and the position of (1000) peaks are barely changed by addition of Si. Based on the shift of (0002) and (1000) peaks, it can be confirmed that addition of Si up to 15 at.% lower the *c*-lattice parameter (Fig. 3(b)) while the *a*-lattice parameter only slightly decreased (Fig. 3(c)). As a result, the lattice parameters ratio (*c/a*) also decrease with increasing Si additions (Fig. 3(d)) and this lattice contraction is likely to be due to the substitution of Al (0.51 Å) that is larger than Si (0.42 Å)^20,21^. Furthermore, higher (0002) intensity that was observed for Si_x_Al_1−x_N with *x* < 0.19 indicate that wurtzite structure is the main component of the thin film which could led to relatively higher *d*_33_. However, lower intensity of (0002) that was exhibited by Si_x_Al_1−x_N with x ≥ 0.19 suggests a decrease in the degree of crystallinity, which explains the lower *d*_33_ generated by these thin films.

### Effect of Si addition as single dopant on chemical state

**Figure 4:**
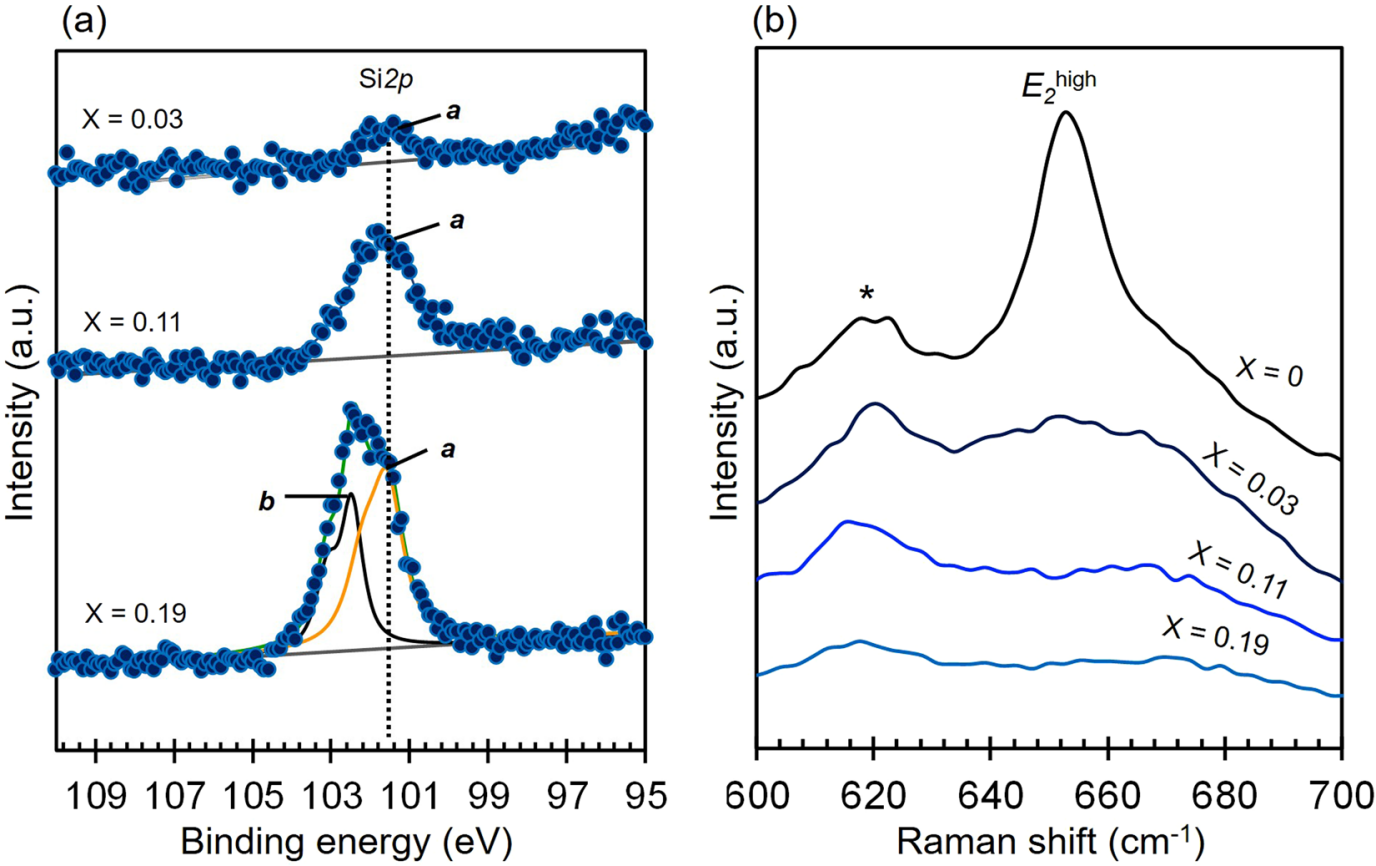
Effect of Si addition on (**a**) Si*2p* spectra and (**b**) Raman spectra of Si_*x*_Al_1−*x*_N, *x* = 0, 0.03, 0.11 and 0.19.

To obtain further insight regarding the effect of Si addition to AlN, changes in binding energy (BE) of Si*2p* was investigated. As depicted in Fig. 4(a), the intensity of Si*2p* spectra increases with increasing Si additions. However, the Si*2p* spectra for lower Si addition (Si_*x*_Al_1−*x*_N with *x* = 0.03& 0.11) consist of a doublet (peak ***a***) while that for higher Si addition (Si_*x*_Al_1−*x*_N with *x* = 0.19) can be deconvoluted into two doublets, i.e. ***a*** and ***b***. The BE of peak ***a*** was found to centered at 101.5 eV while that of peak ***b*** is 102.4 eV. The BE of peak ***a*** is reported to be a typical BE of Si with oxidation state of 4+^22–24^. Peak ***b*** is believed to correspond with different type of Si-N bond in SiN_x_, as reported in^25^. Since the XRD patterns for Si_0.19_Al_0.81_N in Fig. 3(a) has confirmed the presence of additional compound, peak ***b*** can be associated with the presence of this additional compound. The BEs of Al*2p* observed here were within the reported BE for Al^3+^ (Supplementary 2(a)) and the BEs of N*1s* were also consistent with the reported BE for N^3−^ in AlN (Supplementary 2(b))^26–28^. However, the spectra of Al*2p* and N*1s* did not exhibit significant changes upon introduction of Si, except for a slight shift in BE of Al*2p* and N*1s* when Si addition is high (19 at.%).

### Elucidation on mechanism of polarity inversion - effect of intermediate layer on polarity inversion

Since an intermediate layer is often employed to inverse the polarity of a thin film^8–10,13,14^, it is likely that an intermediate layer may have formed prior to the growth of Si_x_Al_1−x_N and cause polarity inversion. Given the smaller size of Si than Al^20,21^, Si is suspected to reach the substrate before Al and form a thin layer of Si_*x*_N_*y*_ as the intermediate layer. If this hypothesis is accurate, the presence of a thin layer of Si_*x*_N_*y*_ is predicted to also capable of inversing the polarity of AlN which is known to be Al-polar. To verify this hypothesis, AlN is used as the top layer and a thin layer of Si_*x*_N_*y*_ was sandwiched between the Si substrate and AlN layer (Si_*x*_N_*y*_/AlN). As shown in Supplementary 3, Si_*x*_N_*y*_/AlN thin film exhibits lower *d*_33_ magnitude compared with AlN and Si_0.11_Al_0.99_N, while the polarity is confirmed to be Al-polar. From this result, it can be inferred that the presence of a thin intermediate layer may not have a strong role in inversing the polarity of the developed thin film.

### Elucidation on mechanism of polarity inversion - effect of Si addition on point defect

The x-ray photoelectron spectroscopy (XPS) investigation has confirmed that Si exist as Si^4+^ and Al exist as Al^3+^ in these examined thin films. Substituting Al^3+^ with Si^4+^ will consequently generate point defects (Si_Al_ and aluminum vacancy (*V*_Al_**′**)) to maintain charge neutrality^29^. In order to confirm the presence of point defects, several thin films were subjected to Raman measurements and the results are presented in Fig. 4(b). It can be seen here that the linewidth of E_2_ (high) at 658 cm^−1^, which is the Raman active mode of AlN^30–32^, becomes broader with increasing Si additions even with low addition of Si (*x* = 0.03). The broadening of Raman bands is reported to be originated from the reduction in phonon lifetime caused by scattering, which can be attributed to the presence of point defects^33–35^. However, a broader Raman line that was observed after addition of Si into AlN is also reported to predominantly correspond with the presence of point defect, i.e. aluminum vacancy (*V*_Al_)^36^. Based on results reported by Klemens *et al*. in^36^ and by considering the strong (0002) peak that can still be observed for Si_0.03_Al_0.97_N (Fig. 3), we believe the main contributor for peak broadening observed at lower concentration range (x = 0.03) is the presence of *V*_Al_. However, the shoulder peak at (0002) that became more prominent with the increase in Si addition (Fig. 3) can be an indication for a decrease in crystallinity. On the other hand, greater Si additions could also lead to the increase in aluminum vacancy (*V*_Al_) concentration. Since lower degree in crystallinity and point defect can be manifested as broader Raman line, both factors are likely to contribute to the peak broadening at higher Si addition (x = 0.11).

Interestingly, incorporating germanium (Ge) or oxygen (O_2_) into AlN which has been proven to capable of inversing the polarity from Al-polar into N-polar^6,17^, is also reported to promote the formation of aluminum vacancy (*V*_Al_**′**), as a compensation for the charge differences^6,37,38^. Therefore, there seems to be a correlation between the presence of aluminum vacancy (*V*_Al_) with polarity inversion.

### Elucidation on mechanism of polarity inversion - effect of Si addition on polarity inversion

Why the presence of aluminum vacancy (*V*_Al_) could encourage polarity inversion? The answer to this question has been elucidated by Youngman and Harris^37,38^, who also found that incorporating O_2_ into AlN could inverse the polarity of AlN. Youngman and Harris proposed that a defect cluster of [*V*_Al_ + O_N_**]** could be formed by addition of O_2_ at lower additions. This cluster has relatively low effect on the stability of the wurtzite structure, thus lower O_2_ addition could not inverse the polarity. However, higher O_2_ concentration increases the concentration of [*V*_Al_ + O_N_**]** defect cluster until it reaches a critical point where Al coordination bond was altered from tetrahedral into octahedral. These octahedral units promote the formation of an inverse domain boundary (IDB), which eventually facilitate the polarity inversion^37,38^. Similar mechanism can be applied for Si-doped-AlN, since the formation of [*V*_Al_ + *n*Si_Al_] defect cluster has been reported to be energetically favorable when Si was doped into AlN^29^. At lower Si concentration range (<1 at.%), the presence of [*V*_Al_ + *n*Si_Al_] cluster at lower concentration may have little effect on the stability of the wurtzite structure, hence the polarity of the thin films at this concentration range was similar with AlN (Al-polar). However, addition of Si at higher concentration range (1–15 at.%) increases the concentration of [*V*_Al_ + *n*Si_Al_] defect cluster which could lead to the transformation of Si coordination bond from tetrahedral to octahedral and eventually form an IDB. This explains the polarity inversion from Al-polar to N-polar by addition of Si in the range of 1–15 at.%.

### Effect of MgSi addition as codopant on the piezoelectric response and the polarity

**Figure 5:**
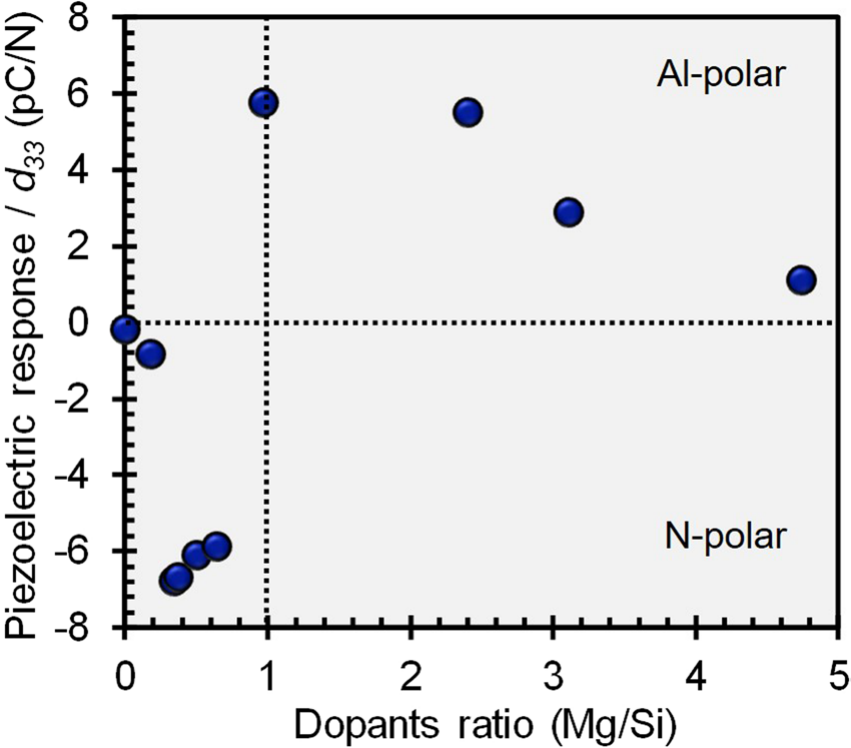
Effect of MgSi codopants ratio on the magnitude and the polarity of the piezoelectric response (*d*_33_) of (MgSi)_x_Al_1−x_N thin films.

As mentioned in the introduction, several reports have revealed that pairing Mg with other elements could be resulted in higher *d*_33_^3,4,18^. Therefore, in order to enhance the *d*_33_ value of Si_x_Al_1−x_N, both Mg and Si was codoped into AlN. For this investigation, the concentration of codopants is fixed in the range of 15–30 at.% and the effect of Mg to Si ratio on the piezoelectric response (*d*_33_) is presented in Fig. 5. It can be seen here that negative *d*_33_ values are  observed when the Mg to Si ratio is less than 1.0, suggesting that the polarity of these thin films is mainly N-polar. However, when MgSi is codoped into AlN with Mg to Si ratio greater than 1, the *d*_33_ values are in positive range which indicate that the polarity of the thin films is predominantly Al-polar. However, the *d*_33_ gradually decreases when Mg/Si ratio is greater than 2.3. From these results, it is confirmed that change of MgSi ratio could switch the polarity of the thin films. However, the enhancement of *d*_33_ was not observed as expected.

**Figure 6:**
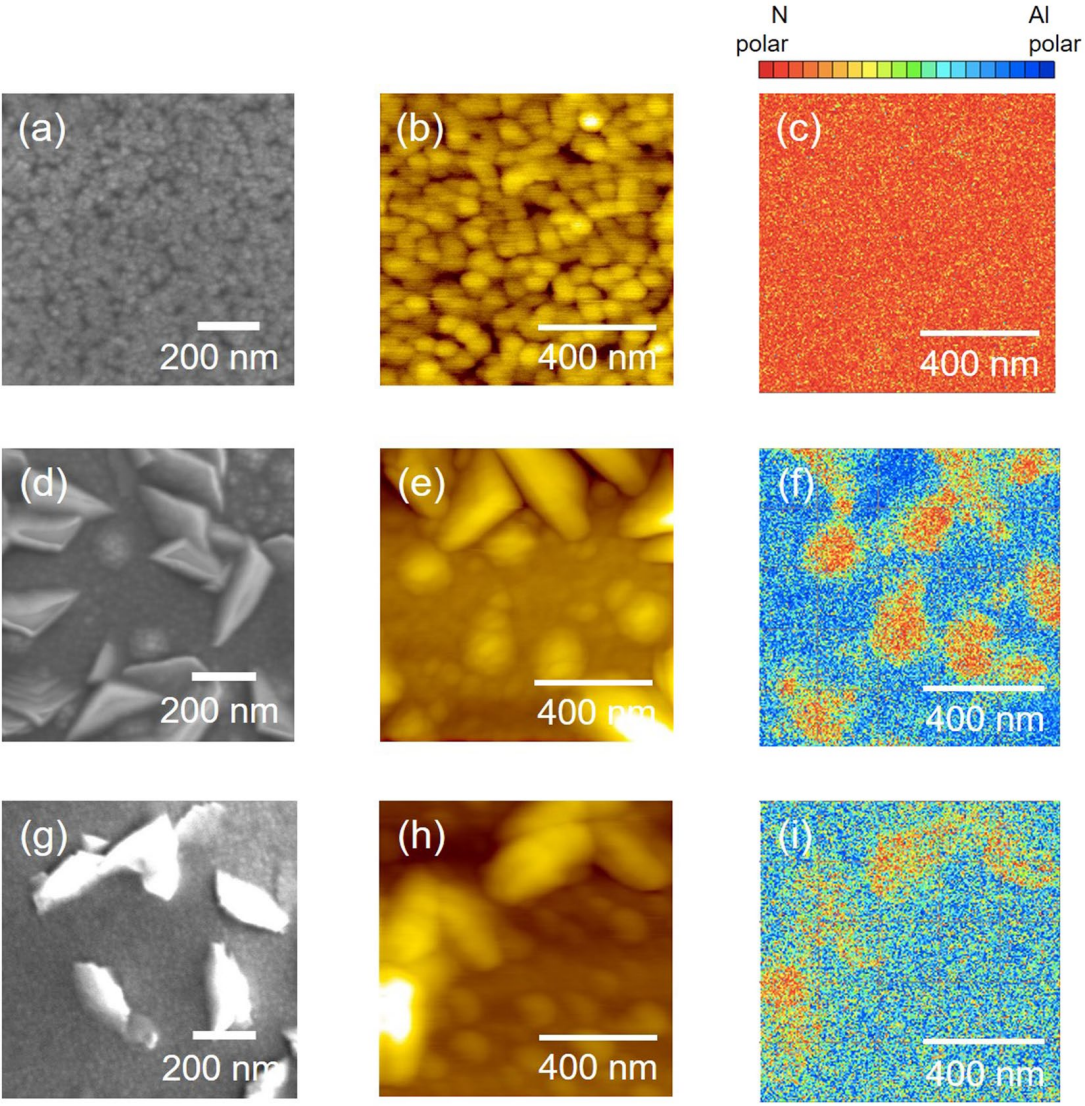
Effect of MgSi codopants ratio on the morphology, topography and polarity of (MgSi)_x_Al_1−x_N thin films with Mg to Si ratio is 0.4 (**a–c**), 1.0 (**d**–**f**) and 2.3 (**g–i**).

For SEM and AFM investigation, three samples are selected as the representative samples, i.e. Mg/Si = 0.4 represents Mg/Si < 1.0, while Mg/Si = 1.0 and 2.3 represent samples with Mg/Si ≥ 1.0. Codoping MgSi with ratio 0.4 yields in particles with size ranging from 40 to 80 nm (Fig. 6(a,b)) and the thin film is found to be mainly N-polar (Fig. 6(c)). A different morphology was observed when the ratio of Mg/Si ≥ 1.0, where it consists of smaller rounded-shaped particles together with greater polygonal-shaped particles and the size of these particles is in the range of 40 to 124 nm (Fig. 6(d,e,g,h)). Although Al-polar component seems to be the main component in thin films with Mg/Si ≥ 1.0, smaller amount of N-polar component can still be observed, and their amount gradually decrease with increasing Mg to Si ratio from 1.0 to 2.3 (Fig. 6(f,i)). Since the amount of Al-polar component is greater than N-polar component, the net polarity of thin films with Mg to Si ratio greater than 1.0 is Al-polar. These results are consistent with the positive *d*_33_ value that was observed for thin films with Mg/Si is 1.0 and 2.3 (Fig. 5).

### Effect of MgSi addition as codopant on the crystal structure

**Figure 7:**
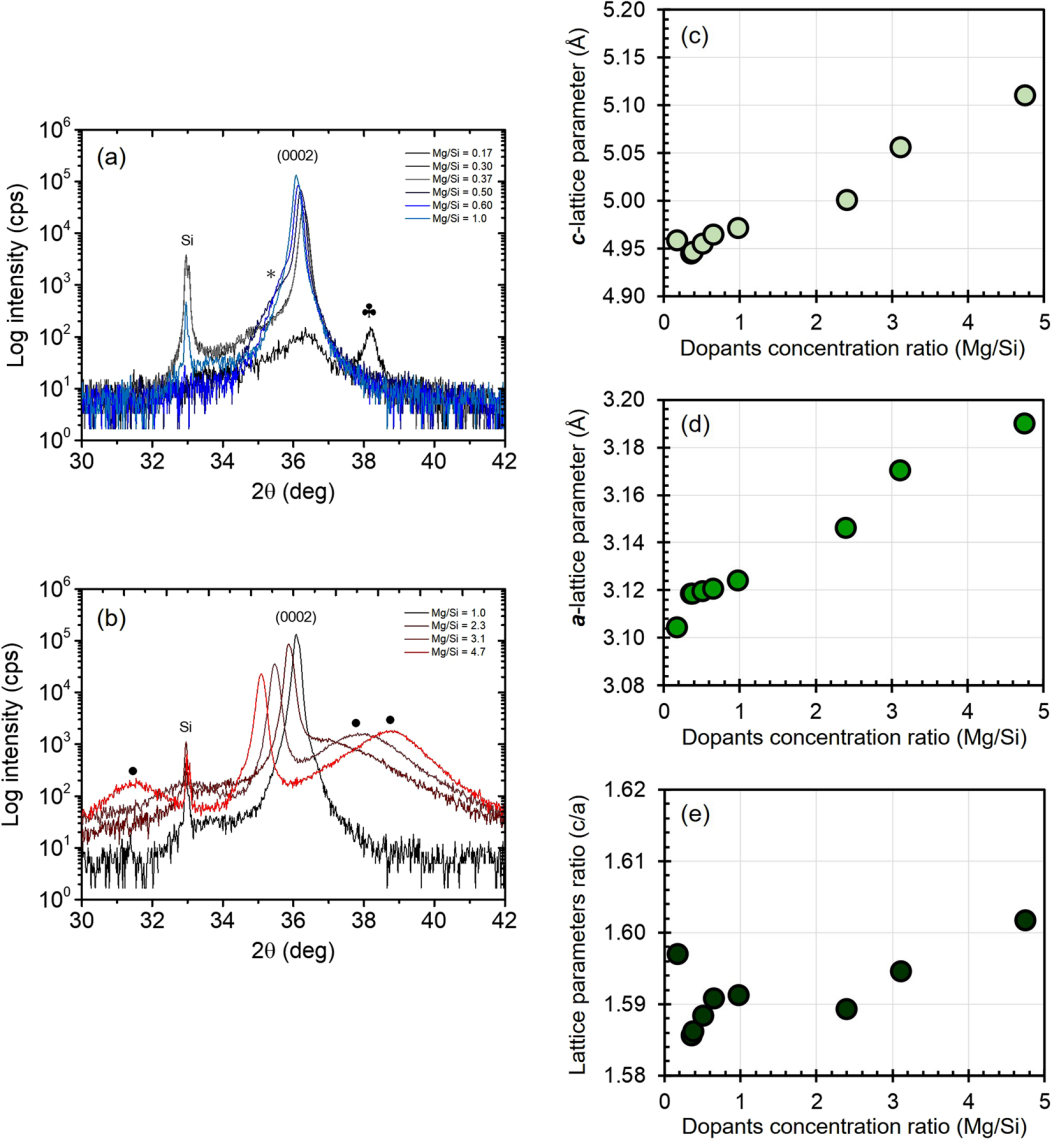
Effect of MgSi addition into AlN on (**a,b**) (0002) peaks and the corresponding lattice parameters ((**c**) *c*-axis and (**d**) *a*-axis)) as well as the (**e**) lattice parameters ratio (*c/a*).

The effect of MgSi ratio is further studied with regards to changes in crystal structure and the corresponding lattice parameters. When Mg/Si ratio <1, the intensity of (0002) peak increases with increasing Mg to Si ratio and it was also found to be accompanied by an unknown shoulder peak (*) (Fig. 7(a)), suggesting the presence of another compound together with wurtzite-structured compound. Similar shoulder peak was also observed for Si_*x*_Al_1−*x*_N (*x* = 0.03–0.15) (Fig. 3(a)). However, a significantly lower (0002) peak and the emergence of another additional peak (♣) with comparable intensity are observed for thin film that has lower Mg to Si ratio (Mg/Si = 0.17), suggesting that wurtzite structured compound is not the main component in this thin film. Thus, lower *d*_33_ was obtained for this sample (Fig. 5). However, having larger Mg concentration than Si (Mg/Si > 1) results in the shift of (0002) peak toward lower degree and also encourage the formation of additional compound (•) (Fig. 7(b)). The presence of (•) was also observed in XRD profile of Mg_0.17_Al_0.83_N, as reported in^39^. When Mg/Si ratio is greater than 2.3, the intensity of (0002) gradually decrease, while the intensity of the additional compound (•) becomes more prominent. The increasing amount of this additional compound may hinder the piezoelectric response of the thin film, hence a lower *d*_33_ was observed for thin films with Mg/Si ratio > 2.3. Meanwhile, peaks that are observed in the in-plane XRD profile for the examined samples are found to be consistent with peaks of wurtzite AlN (ICSD no.34236) (Supplementary 4).

Since (0002) and (1000) peaks are found to shift to lower degree with increasing MgSi ratio, it can be estimated that the *c*-lattice parameter (Fig. 7(c)) and *a*-lattice parameter (Fig. 7(d)) increase with increasing Mg to Si ratio. Consequently, the lattice parameter ratio (*c/a*) gradually increase with increasing Mg to Si ratio (Fig. 7(e)). Changes in lattice parameters obtained here are believed to be due to the substitution of Al with the dominant element (Mg or Si). When Si concentration is greater than Mg (0.2 < MgSi < 1), slight lattice contraction was observed due to greater amount of Si (0.42 Å) replace Al (0.51 Å)^20,21^. On the contrary, lattice expansion that was observed when Mg concentration is greater than Si (Mg/Si > 1) is believed to be due to the greater amount of Mg (0.66 Å) replace Al (0.51 Å)^20,21^.

### Effect of MgSi addition as codopant on chemical state

**Figure 8:**
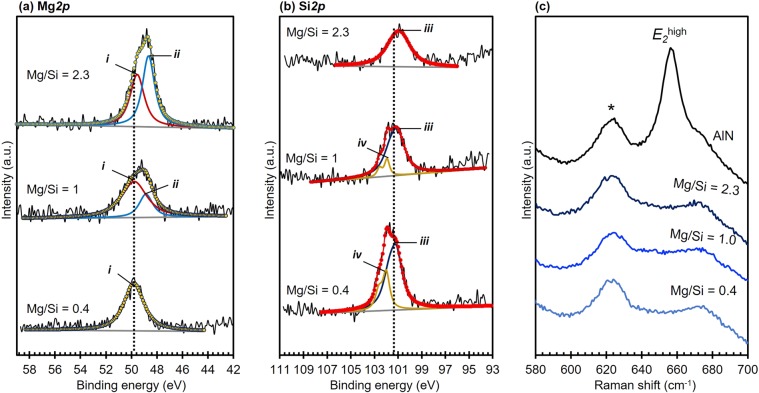
Effect of MgSi addition on (**a**) Mg*2p*, (b) Si*2p* and (**b**) Raman spectra of (MgSi)_x_Al_1−x_N thin films with Mg to Si ratio is 0.4, 1.0 and 2.3.

Changes in binding energy due to MgSi addition are also investigated by subjecting the three samples to XPS measurement. As shown in Fig. 8(a), Mg*2p* spectra for Mg/Si ≥ 1 can be deconvoluted into two peaks ***i*** and ***ii***, while Mg*2p* spectra for Mg/Si = 0.4 only consist of peak ***i***. Peak ***i*** was found to centered at BE of approximately 49.8 eV. These observed BEs were in good agreement with the BEs for Mg^2+^ in AlN (Supplementary 5)^18^. Meanwhile, peak ***ii*** was found to centered at lower BE (48.7 eV) and the area seems to increase with increasing Mg to Si ratio. The presence of such additional peak at similar BE was also observed for Mg_0.17_Al_0.83_N (Supplementary 5). Since XRD patterns for sample with Mg/Si = 2.3 (Fig. 7(b)) confirmed the presence of additional compound, the appearance of peak ***ii*** is believed to correspond with this additional compound.

The effect of Mg to Si ratio on Si*2p* spectra is given in Fig. 8(b). The Si*2p* spectra can be deconvoluted into two doublets for sample with Mg/Si ≤ 1, namely peak ***iii*** and ***iv***, while sample that has Mg/Si ratio of 2.3 only consists of peak ***iii***. Peak ***iii*** is found to centered at approximately 101.2 eV, which is close with the reported BE for Si^4+^ ^22,24,40^. However, the BE of peak ***iv*** which is observed at BE of 102 eV, which has been reported to correspond with different type of Si-N bond (i.e. Si-N-N)^25^ and was also observed in Si*2p* spectra for Si_*0.11*_Al_*0.89*_N (Fig. 4). Since the XRD patterns for sample with Mg/Ta = 0.4 (Fig. 7(a)) suggested the presence of an additional compound (*), these additional doublets are believed to correspond with the presence of this additional compound. However, changes in Mg to Si ratio does not seem to significantly affect the BEs of Al*2p* and N*1s*, since they are in close agreement with the observed BE for Al^3+^ in AlN (Supplementary 6(a))^26–28^ and for N^3-^ in AlN, respectively (Supplementary 6(b))^26–28^. However, the width of N*1s* spectra is slightly affected by Mg/Si ratio, which might be correlate with the presence of multiple nitride compounds in the thin films, as have been also indicated by Mg*2p* and Si*2p* spectra. The presence of multiple nitride compounds could also indicate that the solubility limit of MgSi in AlN to maintain a stable wurtzite structure may be lower than the examined concentration range (15–30 at.%). Excess of Mg and/or Si could also form nitride compounds, in addition to (MgSi)_*x*_Al_1−*x*_N.

### Effect of MgSi addition as codopant on the formation of defect

The presence of thin intermediate layer has been confirmed in the previous section to have smaller influence in inversing the polarity than point defects, hence effect of MgSi addition on polarity was investigated with respect to the presence of point defects (*V*_Al_ or *V*_N_) via Raman investigation. The effect of different Mg to Si ratio on the Raman band of E_2_(high) at 658 cm^−1^ is given in Fig. 8(c), where incorporating MgSi at ratio of 0.4, 1.0 and 2.3 result in broader E_2_(high) linewidth. Since XPS results have suggested that the solubility limit of MgSi may be lower than the examined concentration range (15–30 at.%) (Fig. 8(a,b)), an excess of either Si and/or Mg would form defects which could be manifested as broader Raman bands. Large excess of Si in samples with Mg/Si = 0.4 would lead to greater amount of Si_*x*_Al_1−*x*_N than (MgSi)_*x*_Al_1−*x*_N. The formation of Si_*x*_Al_1−*x*_N is confirmed to be energetically favorable when followed by the formation of *V*_Al_^29^, which can be contributed to the broader linewidth of E_2_(high), as observed for samples with Mg/Si = 0.4. However, broader Raman bands are also observed for sample with Mg to Si ratio ≥ 1.0. Although samples that have Mg/Si ≥ 1.0 is believed to mainly consists of (MgSi)_*x*_Al_1−*x*_N, the lower solubility limit of MgSi might result in excess of both Mg and Si. Excess of Mg will form Mg_*x*_Al_1−*x*_N and create nitrogen vacancy (*V*_N_)^41^. Broader Raman bands due to addition of Mg or Cu as single dopant for AlN has also been reported elsewhere^41,42^. Meanwhile, the excess of Si will form Si_*x*_Al_1−*x*_N and *V*_Al_, which has been reported to affect the Raman bands^36^. Thus, the presence of multiple defects namely nitrogen (*V*_N_) and aluminum (*V*_Al_) vacancies in sample with Mg/Si ≥ 1.0 could cause broader Raman band (Fig. 8(c)).

### Effect of MgSi addition on polarity inversion

Incorporating both Mg and Si into AlN in different ratio has been confirmed to alter the composition of compounds that construct the thin films, and this could affect the net polarity of the thin films. Large excess of Si in samples with Mg/Si < 1.0 would result in greater amount of Si_*x*_Al_1−*x*_N than (MgSi)_*x*_Al_1−*x*_N and consequently followed by the formation of high concentration of *V*_Al_ which form defect cluster of [*V*_Al_ + *n*Si_Al_]. Similar with the case of Si-doped-AlN, high concentration of [*V*_Al_ + *n*Si_Al_] defect cluster could transform Si coordination from tetragonal to octahedral, hence an IDB that facilitate polarity inversion can be created. Meanwhile, a wurtzite (MgSi)_*x*_Al_1−*x*_N could maintain its stability without creating *V*_N_ or *V*_Al_, hence the polarity of wurtzite (MgSi)_*x*_Al_1−*x*_N is expected to be similar with AlN (Al-polar). Thus, since Si_*x*_Al_1−*x*_N exists in greater amount than (MgSi)_*x*_Al_1−*x*_N, having Mg to Si ratio less than 1 resulted in thin films with N-polarity.

On the contrary, thin films with Mg/Si ratio ≥ 1 have been proven to mainly composed of Al-polar components and smaller amount of N-polar components. As has been mentioned above, the low solubility limit of MgSi made addition of MgSi at the examined concentration range yielded in excess of Mg and Si. The excess of Mg will form Mg_*x*_Al_1−*x*_N as well as *V*_N_ and their coexistence has been proven to result in thin film with Al-polarity^39^. Meanwhile, the presence of smaller amount of N-polar components is believed to correspond with the existence of Si_*x*_Al_1−*x*_N as a product from the excess of Si, whose formation could induce defect cluster of [*V*_Al_ + *n*Si_Al_]^29^ that lead to polarity inversion. Since the excess of Si exist in smaller amount, the polarity inversion also occurs locally. Greater number of Al-polar compounds (which are believed to consist of (MgSi)_*x*_Al_1−*x*_N and Mg_*x*_Al_1−*x*_N) than that of the N-polar compound (Si_*x*_Al_1−*x*_N) yielded a net polarity of Al-polar for these thin films. However, increasing MgSi ratio will reduce the excess of Si, hence a gradually lower amount of N-polar component was observed with increasing Mg/Si ratio.

## Conclusions

Introducing 1–15 at.% Si into AlN has been proven to inverse the polarity from Al-polar to N-polar. Addition of Si at that concentration range could maintain a stable wurtzite structure while changing the lattice parameters and its ratio. Inserting a thin intermediate layer of Si_*x*_N_*y*_ was unable to inverse the polarity of AlN from Al-polar to N-polar, whereas  the presence of *V*_Al_ which was induced by the addition of Si into AlN seems to strongly affect the polarity inversion. The presence of high concentration of defect cluster of [*V*_Al_ + Si_Al_] in Si_*x*_Al_1−*x*_N is believed to transform the coordination of Si from tetragonal to octahedral, which facilitate the formation of an inverse domain boundary (IDB) that eventually lead to the polarity inversion from Al-polar to N-polar.

For the case of MgSi, codoping Mg and Si into AlN at different ratio resulted in multiple nitride compounds that eventually yielded in different net polarity. The domination of Si_*x*_Al_1−*x*_N in thin films with Mg/Si < 1 is believed to contribute in generating net polarity of N-polar. Meanwhile, the presence of Al-polar compounds ((MgSi)_*x*_Al_1−*x*_N and Mg_*x*_Al_1−*x*_N) as the dominant component in the thin films with Mg/Si ≥ 1 resulted in net polarity of Al-polar. Considering importance of *V*_Al_ and *V*_N_ in inversing the polarity, further and detailed investigation is required to gain deeper understanding regarding the role those point defects in polarity inversion. Such knowledge would be beneficial to control the polarity of nitride-based thin films and to develop high performance electronic devices.

## Methods

### Fabrication of thin films

The thin film was fabricated by utilizing a radio frequency (RF) sputtering system that is equipped with triple targets, namely Al (99.999%, Raremetalic, Japan), Si (99.99%, Raremetalic, Japan) and Mg (99.99%, Raremetalic, Japan). The concentration of dopants was adjusted by controlling the output power of the target during sputtering process. The nitride thin films were directly deposited on the surface of Si (100) wafer (square-shaped with size of 17 mm × 17 mm). Before the sputtering process was began, the sputtering chamber was evacuated to a pressure of less than 1 × 10^−5^ Pa. The deposition process of the thin film was conducted for 4 h at temperature of 400 °C, deposition pressure of 0.35 Pa and N_2_ concentration was fixed at 50 vol.% (total gas (Ar + N_2_) flow was kept at 10 ccm). To study the effect of intermediate layer, a thin Si_x_N_y_ as the intermediate layer was fabricated by sputtering Si target for 1h under the same deposition parameters prior to AlN deposition.

### Characterization of thin films

The piezoelectric response (*d*_33_) as well as the polarity was investigated by clamping the sample and applying a low frequency force (0.25 N at 110 Hz) using a Piezometer system (Piezotest PM300, UK). Al electrode were deposited on the surface of the thin film as the top electrode. Since Si wafer is a conductive material, a bottom electrode is not necessary. The measurements were conducted under low range mode, which capable to examine *d*_33_ in the range of 1–100 pC/N with accuracy of ±2% ±0.1 pC/N. A correction of the obtained *d*_33_ values was not performed. The concentration of dopants was determined by an energy dispersive x-ray spectroscopy (EDX) (Horiba, Japan). The polarity distribution was examined using piezoresponse force microscopy (PFM) (SPI-3800N, Seiko Instr. Inc., Japan) with modulation frequency of 10 kHz and a driving voltage of 30 V was applied to the tip. The crystal structure of the obtained films was evaluated by subjecting each sample to measurement using an out-of-plane x-ray diffractometer (XRD, RINT-TTR III, Rigaku, Japan). Samples were also subjected to in-plane XRD measurement using SmartLab XRD with Cu Kα (Rigaku, Japan). The $c$-lattice parameter was determined from the (002) reflection from out-of-plane XRD measurements and the $a$-lattice parameter was analyzed from (100) reflection that was obtained by the in-plane XRD measurements using the following formula:

$$\frac{1}{({d}_{hkl}^{2})}=\frac{4}{3}\left(\frac{{h}^{2}+hk+{k}^{2}}{{a}^{2}}\right)+\frac{{l}^{2}}{{c}^{2}}$$

where $h$, $k$, $l$ are miller indices, $c$ and $a$ are lattice constant for $c$-axis and $a$-axis, respectively and $d$ is the spacing of (${hkl}$) planes. The morphology of the thin film was studied using field emission scanning electron microscopy (FE-SEM, JSM-7001F, JEOL, Japan), operated at 5 kV. The x-ray photoelectron spectroscopy (XPS) measurements were performed using KRATOS Axis 165 (Shimadzu, Japan) with monochromatic Al Kα source for excitation (12 kV and 2 mA) under high vacuum (1.18 × 10^–6^ Pa). The C*1s* line of 284.6 eV was used as reference to calibrate the binding energy. The presence of defect was investigated by using Raman spectroscopy (Nanofinder 30, Tokyo Instruments, Japan) using laser wavelength of 532 nm.

## Supplementary Information

Supplementary Information.
