# Supplementary material for: Polarity Inversion of Aluminum Nitride Thin Films by using Si and MgSi Dopants
Source: Sci Rep. 2020 Mar 9;10:4369. doi: 10.1038/s41598-020-61285-8 (PMC7062775; doi:10.1038/s41598-020-61285-8)

Effect of incremental Si addition into AlN on the in-plane XRD spectra of the resulting thin films.

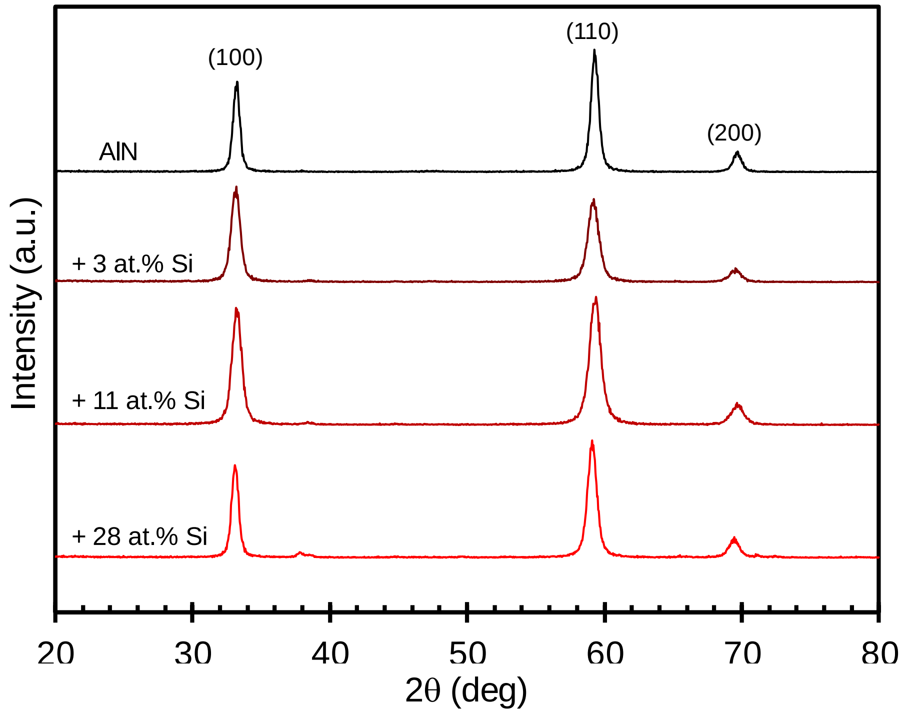

Effect of incremental Si addition into AlN on the (a) Al*2p* and (b) N*1s* spectra of the resulting thin films.

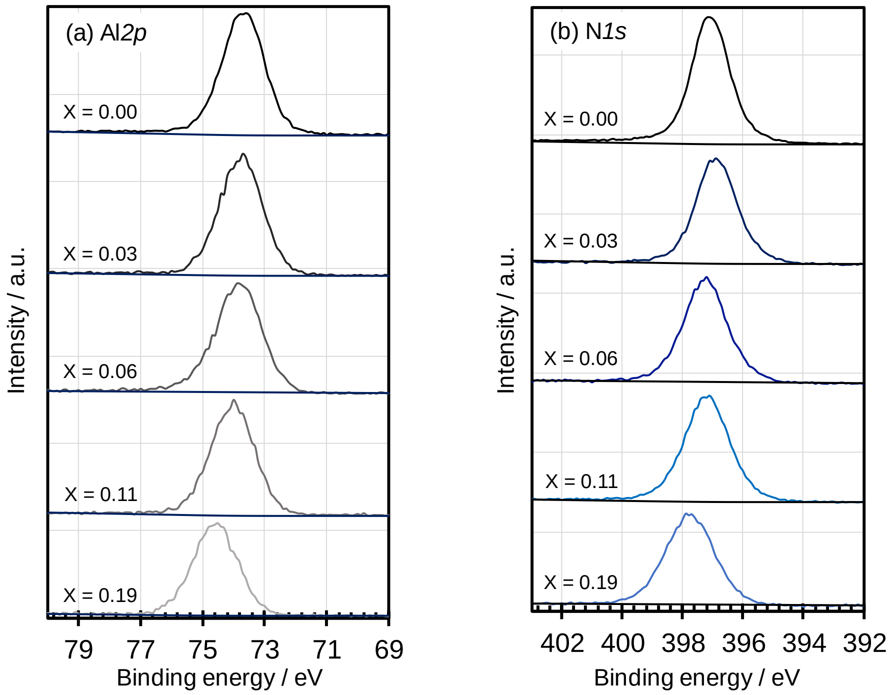

Table 1 Comparison of piezoelectric response (d_{33}) as well as the polarity of different thin films with or without intermediate layer.

**Thin film on Si (100) substrate**	**d_{33} (pC/N)**	**polarity**
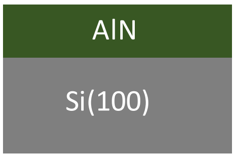 AlN	6.8	Al polar
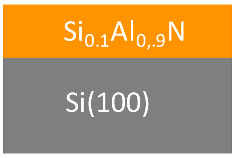 $Si_{0.1}Al_{0.9}N$	-6.3	N polar
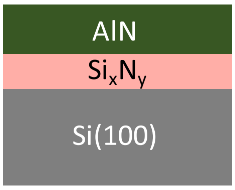 Si_xN_y/AlN	1.8	Al polar

Effect of different MgSi ratio on the in-plane XRD spectra of the resulting thin films.

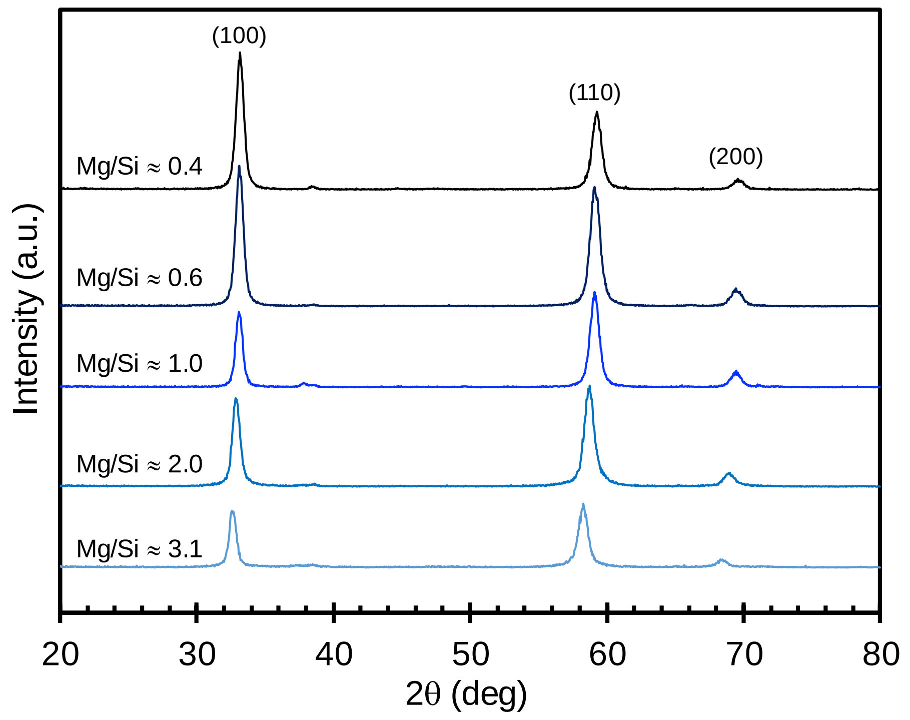

Effect of Mg addition as a single dopant into AlN on Mg*2p* spectra of the resulting thin films.

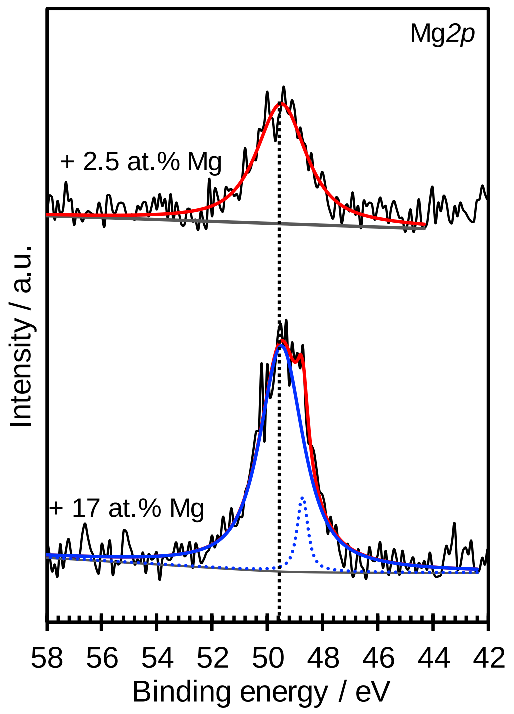

Effect of MgSi ratio into AlN on the (a) Mg*2p* and (b) N*1s* spectra of the resulting thin films.

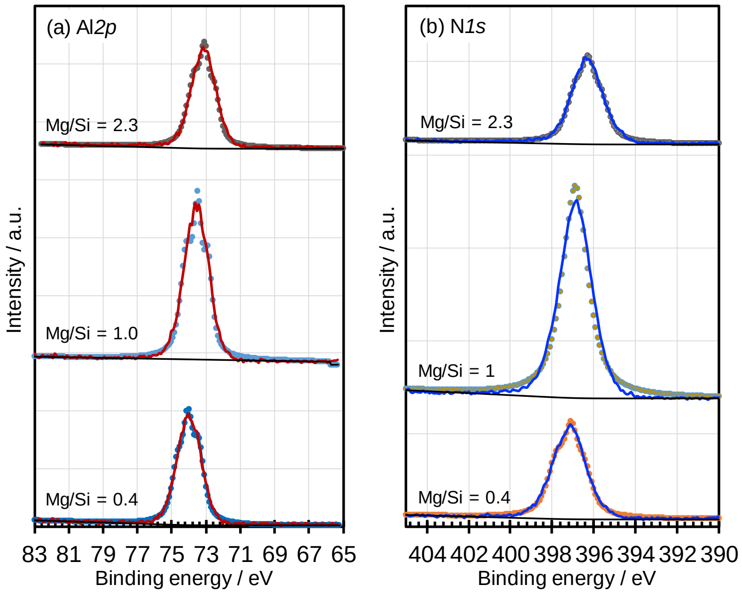

Supplement: Supplementary file 1 — Supplementary Information. [file 41598_2020_61285_MOESM1_ESM.pdf]
